# Supplementary material for: Investigating novel Streptomyces bacteriophage endolysins as potential antimicrobial agents
Source: Microbiol Spectr. 2024 Nov 21;13(1):e01170-24. doi: 10.1128/spectrum.01170-24 (PMC11705968; doi:10.1128/spectrum.01170-24)
Supplement: File S2 — List of the 250 putative endolysins with protein information and GenBank accession number. [file spectrum.01170-24-s0002.pdf]

Additional File 2

Additional File 2. 250 putative endolysins analyzed in this study. Identified catalytic domains include amidase (IPR002502), peptidoglycan recognition protein (PGRP; IPR006619), cysteine, histidine-dependent amidohydrolases/peptidases (CHAP; IPR007921), and O-Glycosyl hydrolases (IPR018077). Predicted binding domains are LysM (IPR018392), CW-7 (IPR013168), PG-bd-like (IPR002477), and SH3.

No	Endolysins	Genbank/pham Accession no.	Phage Cluster	Subcluster	AA length	Mass (kDa)	Predicted Function (by SEA-PHAGES)	Notes
**Amidase-LysM**			**20.0%**					
1	>SPB78 gp21	NZ_ACEU00000000.1	BA	None	275	29.0	N/A	
2	>Nabi gp26	AWN07320.1	BD	BD1	364	39.0	LysM-like endolysin	
3	>Toma gp26	AWN07621.1	BD	BD1	362	38.7	LysM-like endolsyin	
4	>Asten gp25	QAY17707.1	BD	BD1	362	38.5	LysM-like endolysin	
5	>Goby gp26	AWN07545.1	BD	BD1	362	38.7	LysM-like endolysin	
6	>Lika gp26	AGM12049.1	BD	BD1	364	39.1	N/A	
7	>Whatever gp25	QFP95192.1	BD	BD1	362	38.5	lysin A	
8	>Godpower gp27	AOQ27003.1	BD	BD1	364	39.1	endolysin	
9	>Dattran gp27	ATE85131.1	BD	BD1	362	38.7	endolysin	
10	>Lorelei gp26	AOQ26925.1	BD	BD1	364	39.0	endolysin	
11	>Danzina gp27	YP_009592392.1	BD	BD1	362	38.7	LysM	
12	>Brataylor gp28	AOQ27076.1	BD	BD1	364	38.9	endolysin	
13	>Zemlya gp 27	AGM12202.1	BD	BD1	362	38.7	N/A	
14	>Sujidade gp27	AGM12125.1	BD	BD1	362	38.8	N/A	
15	>Yasdnil gp25	YP_010056432.1	BD	BD1	362	38.5	LysM-like endolysin	
16	>TuanPN gp25	QDK03196.1	BD	BD1	362	38.5	LysM-like endolysin	
17	>OzzyJ gp26	AVE00407.1	BD	BD1	362	38.5	LysM-like endolysin	

(table continues)

No	Endolysins	Genbank/pham Accession no.	Phage Cluster	Subcluster	AA length	Mass (kDa)	Predicted Function (by SEA-PHAGES)	Notes
**Amidase-LysM**			**20.0%**					
18	>Rana gp26	AWN07244.1	BD	BD1	364	39.0	LysM-like endolysin	
19	>Maneekul gp25	AWN07394.1	BD	BD1	362	38.5	LysM-like endolysin	
20	>Celeste gp26	ATE85054.1	BD	BD1	362	38.7	endolysin	
21	>Teutsch gp44	QAX95780.1	BE	BE1	474	50.8	LysM-like PGBD	
22	>Mildred21 gp42	YP_009610583.1	BE	BE1	336	36.4	LysM-like PGBD	
24	>Paradiddles gp40	YP_009611036.1	BE	BE1	471	50.4	LysM-like PGBD	
25	>Braelyn gp42	YP_010103934.1	BE	BE1	471	50.5	LysM-like PGBD	
26	>Egole gp45	YP_010101465.1	BE	BE1	474	50.7	LysM-like PGBD	
27	>Tribute gp42	QGH78234.1	BE	BE1	474	50.5	LysM-like PGBD	PGRP like amidase
28	>Sushi23 gp44	ASR76476.1	BE	BE1	473	50.7	LysM-like PGBD	
29	>Samisti12 gp44	YP_009611482.1	BE	BE1	474	50.8	LysM-like PGBD	
30	>MulchMansion gp41	QNO12465.1	BE	BE1	340	36.7	Endolysin	30% Amidase (4BOL), 22-28% LysM (4B9H and 4S3K)
31	>LilMartin gp41	QNN98291.1	BE	BE1	340	36.7	endolysin	See MulchMansion
32	>Bmoc gp42	YP_010107442.1	BE	BE1	340	36.8	endolysin	
33	>Peebs gp43	YP_009611255.1	BE	BE1	474	50.8	LysM-like PGBD	29% Amidase (4BOL), 27% LysM (4UZ2)
34	>LukeCage gp45	YP_009839969.1	BE	BE2	331	36.0	LysM-like PGBD	
35	>StarPlatinum gp45	YP_009839482.1	BE	BE2	331	36.0	LysM-like PGBD	
36	>Yaboi gp46	YP_009841182.1	BE	BE2	297	32.1	LysM-like PGBD	
37	>Genie2 gp45	QAY08707.1	BE	BE2	297	32.1	LysM-like PGBD	
38	>BoomerJR gp46	QAY12697.1	BE	BE2	297	32.1	LysM-like PGBD	
39	>Wofford gp43	YP_009839732.1	BE	BE2	339	37.0	LysM-like PGBD	
40	>MindFlayer gp44	QPL13684.1	BE	BE2	293	31.6	Endolysin	
41	>SparkleGoddess gp22	AXH68737.1	BK	BK1	338	36.4	LysM-like endolysin	
42	>Limpid gp21	QGH79354.1	BK	BK1	338	36.3	LysM-like endolysin	
43	>Gilson gp25	YP_009842488.1	BK	BK1	338	36.5	LysM-like endolysin	
44	>Comrade gp22	YP_009840815.1	BK	BK1	337	36.3	LysM-like endolysin	

45	>Annadreamy gp21	YP_009838987.1	BK	BK1	338	36.3	LysM-like endolysin	

(table continues)

No	Endolysins	Genbank/pham Accession no.	Phage Cluster	Subcluster	AA length	Mass (kDa)	Predicted Function (by SEA-PHAGES)	Notes
**Amidase-LysM**			**20.0%**					
46	>Blueeyedbeauty gp23	YP_009839218.1	BK	BK1	338	36.4	LysM-like endolysin	
47	>Beuffert gp20	QOI67422.1	BK	BK1	350	37.3	LysM-like endolysin	Align with 5JCE (26% identity)
48	>Wakanda gp17	QIN94010.1	BK	BK2	338	36.4	lysin, LysM-like	
49	>Muntaha gp17	QIN94574.1	BK	BK2	336	36.3	lysin, LysM-like	Amidase (Autolysin: 4KNK_A residue 46-160 aligned with Attoomi residue 10-135 with 99.13% probability, 19% identity) HHpred
50	>Attoomi gp20	YP_009796801.1	Singleton	-	348	37.4	LysM-like endolysin	
**PGRP/Amidase-PG-bd-like**			**39.6%**					
1	>VWB gp22	AAR29740.1	BA	None	275	29.5	N/A	
2	>Mojorita gp21	APD18698.1	BC	BC1	260	28.0	endolysin	
3	>Picard gp21	YP_009788224.1	BC	BC1	260	28.0	endolysin	
4	>SV1 gp21	AFU62161.1	BC	BC1	315	33.4	putative endolysin	
5	>ToastyFinz gp21	ARB11443.1	BC	BC1	355	37.8	endolysin	
6	>Raleigh gp22	APD18773.1	BC	BC2	360	37.8	endolysin	
7	>Darolandstone gp22	YP_009812933.1	BC	BC2	361	37.5	lysin A	
8	>Austintatious gp21	YP_009819793.1	BC	BC3	313	33.2	endolysin	
9	>Ididsumtinwong gp21	YP_009788115.1	BC	BC3	313	33.2	endolysin	
10	>PapayaSalad gp21	YP_009788170.1	BC	BC3	313	33.2	endolysin	
11	>Bioscum gp21	APD18718.1	BC	BC3	313	33.3	endolysin	
12	>Esperer gp26	ATE85206.1	BD	BD1	317	33.8	endolysin	
13	>BryanRecycles gp27	ATE84980.1	BD	BD1	313	33.2	endolysin	
14	>Oliynyk gp27	ATE85357.1	BD	BD1	313	33.2	endolysin	
15	>BeardedLady gp27	ATE84905.1	BD	BD1	317	33.8	endolysin	
16	>Jash gp27	ATE85330.1	BD	BD1	313	33.2	endolysin	
17	>Nanodon gp28	YP_009287812.1	BD	BD1	314	32.9	endolysin	

18	>Aaronocolus gp 26	YP_009616451.1	BD	BD1	317	33.8	endolysin	

(table continues)

No	Endolysins	Genbank/pham Accession no.	Phage Cluster	Subcluster	AA length	Mass (kDa)	Predicted Function (by SEA-PHAGES)	Notes
**PGRP/Amidase-PG-bd-like**			**39.6%**					
19	>Izzy gp27	YP_009215405.1	BD	BD1	313	33.2	Endolysin	
20	>Hydra gp28	AKY03559.1	BD	BD1	317	33.8	LysM	
21	>Lannister gp28	YP_009200968.1	BD	BD1	317	33.6	endolysin	
22	>Caliburn gp26	YP_009207123.1	BD	BD1	317	33.8	endolysin	
23	>Phettuccine gp26	QGJ91543.1	BD	BD1	317	33.8	lysin A	same as esperer
24	>Rusticus gp27	QDK03958.1	BD	BD1	313	33.2	LysM-like endolysin	same as esperer
25	>Leviticus gp26	QDK03391.1	BD	BD1	317	33.8	LysM-like endolysin	PRPG 34%, AmpDh2 21%
26	>Nerdos gp26	QAY17844.1	BD	BD1	317	33.8	LysM-like endolysin	same as esperer
27	>Indigo gp25	QAY17301.1	BD	BD1	317	33.8	LysM-like endolysin	
28	>Bovely gp26	QAY17229.1	BD	BD1	317	33.8	LysM-like endolysin	Amidase-lysM-PG-bdlike, PRPG 30%, AmpDh2 22% high coverage.
29	>Eddasa gp27	AWN07470.1	BD	BD1	313	33.2	LysM-like endolysin	
30	>Ozzie gp26	ATE85431.1	BD	BD1	317	33.8	endolysin	same as esperer
31	>Paedore gp26	YP_010055892.1	BD	BD2	358	37.6	Lysin	
32	>R4 gp26	AFU62079.1	BD	BD2	356	37.1	putative endolysin	same as ELB20
33	>ELB20 gp25	AFO10891.1	BD	BD2	356	37.1	N/A	
34	>Hank144 gp27	YP_010055079.1	BD	BD2	350	37.4	Lysin A	
35	>Tefunt gp26	YP_010055394.1	BD	BD2	317	34.0	Lysin	
36	>Diane gp26	YP_010055236.1	BD	BD2	317	34.0	Lysin	
37	>Haizum gp26	AXH70230.1	BD	BD2	317	34.1	Lysin A	
38	>Animus gp27	QFG10695.1	BD	BD2	358	38.1	Lysin	
39	>Janus gp27	QAY15931.1	BD	BD2	358	38.1	Lysin A	
40	>Nishikigoi gp26	QAY15767.1	BD	BD2	317	34.1	Lysin A	
41	>Amethyst gp26	YP_010055315.1	BD	BD2	316	34.0	Lysin	
42	>SqueakyClean gp27	ATI18890.1	BD	BD2	358	38.1	Lysin	
43	>phiCAM gp28	YP_009592107.1	BD	BD3	226	24.3	N/A	
44	>Yosif gp28	YP_010054670.1	BD	BD3	317	34.1	Lysin	
45	>Verse gp27	AKY03857.1	BD	BD3	323	34.6	endolysin	See Amela
46	>Amela gp27	AKY03782.1	BD	BD3	323	34.5	endolysin	34% PRPG (4C8I)/Amidase (2F2L), 20%AmpDh2 (4BOL)

47	>phiHau3 gp28	YP_006906203.1	BD	BD4	350	37.2	putative endolysin	
48	>Urza gp28	QFG10491.1	BD	BD6	328	34.9	lysin A	

(table continues)

No	Endolysins	Genbank/pham Accession no.	Phage Cluster	Subcluster	AA length	Mass (kDa)	Predicted Function (by SEA-PHAGES)	Notes
**PGRP/Amidase-PG-bd-like**			**39.6%**					
49	>Celia gp28	YP_010054592.1	BD	BD6	328	34.9	LysM-like endolysin	
50	>Daubenski gp45	YP_010104808.1	BE	BE1	298	31.9	LysM-like PGBD	
51	>Wipeout gp44	QGH74290.1	BE	BE2	293	31.6	LysM-like PGBD	
52	>TomSawyer gp44	QGH78931.1	BE	BE2	293	31.6	LysM-like PGBD	
53	>Starbow gp44	AXH66553.1	BE	BE2	293	31.6	LysM-like PGBD	
54	>Birchlyn gp43	QDF17219.1	BE	BE2	293	31.6	LysM-like PGBD	
55	>Karimac gp45	YP_009840217.1	BE	BE2	293	31.6	LysM-like PGBD	
56	>IchabodCrane gp43	QFP97359.1	BE	BE2	293	31.6	LysM-like PGBD	
57	>Bordeaux gp44	QGH79816.1	BE	BE2	293	31.6	LysM-like PGBD	Same as Karimac gp45
58	>HaugeAnator gp2	AUG87329.1	BF	None	253	28.0	Lysin A	
59	>ZooBear gp2	AUG87585.1	BF	None	253	28.0	Lysin A	
60	>ToriToki gp 2	AUG87521.1	BF	None	254	28.1	Lysin A	
61	>Romero gp2	AUG87457.1	BF	None	254	28.1	Lysin A	
62	>Percastrophe gp2	AUG87393.1	BF	None	254	28.1	Lysin A	
63	>Olicious gp2	AZF95812.1	BF	None	253	28.0	Lysin A	
64	>Immanuel3 gp2	YP_009836121.1	BF	None	254	28.1	Lysin A	
65	>Geostin gp2	QEA11225.1	BF	None	257	27.9	Lysin A	Same as Fabian
66	>Fabian gp2	QFP94721.1	BF	None	255	27.7	Lysin A	
67	>FlowerPower gp2	YP_009838059.1	BF	None	257	27.9	Lysin A	Same as Fabian
68	>WRightOn gp3	YP_009835995.1	BF	None	259	28.3	Lysin A	
69	>Manuel gp2	YP_009836058.1	BF	None	233	25.5	Lysin A	
70	>Salete gp27	AWN08458.1	BG	None	420	44.1	Lysin	
71	>BayC gp27	AWN08387.1	BG	None	420	44.1	Lysin	
72	>Abt2graduatex2 gp28	ATN93711.1	BG	None	425	44.8	Lysin	
73	>BabyGotBac gp27	APZ82195.1	BG	None	420	44.1	Lysin	
74	>Maih gp27	ALY07277.1	BG	None	420	44.1	Lysin	

75	>Xkcd426 gp33	AMD42774.1	BG	None	335	35.6	Lysin	
76	>TP1604 gp27	AKA61765.1	BG	None	420	44.1	Lysin	

(table continues)

No	Endolysins	Genbank/pham Accession no.	Phage Cluster	Subcluster	AA length	Mass (kDa)	Predicted Function (by SEA-PHAGES)	Notes
**PGRP/Amidase-PG-bd-like**			**39.6%**					
77	>YDN12 gp29	AKA61696.1	BG	None	422	44.4	Lysin	
78	>Mischief19 gp36	QBZ73521.1	BG	None	321	34.2	Lysin A	
79	>Dubu gp18	QDH92123.1	BJ	None	312	34.1	lysin A, N-acetylmuramoyl-L-alanine amidase domain	
80	>phiSASD1 gp40	YP_003714747.1	BJ	None	278	29.6	endolysin	22% Amidase (1YB0), 27% PG-bd-like 5NM7
81	>Moab gp22	QIQ62907.1	BK	BK1	299	31.9	Lysin A	same as IchabodCrane gp43
82	>Satis gp118	AXH66279.1	BM	None	361	38.0	lysin A	AmphD2, 30% identity with high coverage
83	>JustBecause gp116	AYD81285.1	BM	None	361	38.2	lysin A	same as Kradal
84	>Kradal gp118	QBZ72016.1	BM	None	361	38.0	lysin A	looks like amidase-lysm, 28% identity to AmpDh2 with high coverage
85	>Yara gp25	AVP41359.1	BN	None	307	33.9	lysin	
86	>Gibson gp28	QAX92974.1	BN	None	309	32.8	endolysin	
87	>Wentworth gp29	AVP41468.1	BN	None	305	32.6	lysin	
88	>Forthebois gp35	YP_010084058.1	BO	None	258	27.0	LysM-like endolysin	
89	>WheeHeim gp36	YP_010084094.1	BO	None	261	27.3	LysM-like endolysin	
90	>Hiyaa gp30	YP_009818466.1	BQ	None	324	35.9	LysM-like endolysin	
91	>BRock gp39	YP_009831765.1	Singleton	None	310	34.5	N/A	Amidase (AmiE 3LAT 20%), PG-bd-like 31% 5TV7
92	>Chymera gp20	AMS01579.1	Singleton	None	291	31.6	Lysin	
93	>Zuko gp35	QEQ93613.1	Singleton	None	317	34.4	lysin A, N-acetylmuramoyl-L-alanine amidase domain	
94	>pZL12 gp44	ACX71121.1	Singleton	None	312	33.0	N/A	
95	>phiSAV gp25	BAC73210.1	Singleton	None	257	27.7	N/A	
96	>Gilgamesh gp88	QFG13280.1	Singleton	None	351	37.4	lysin A, N-acetylmuramoyl-L-alanine amidase domain	
97	>Kromp gp25	AYD81626.1	Singleton	None	308	33.1	LysM-like endolysin	
98	>Ibantik gp85	AWN05307.1	Singleton	None	351	37.4	Lysin A	
99	>mu1/6 gp19	YP_579222.1	Singleton	None	393	42.1	N/A	Amidase aligned with AmpDh2 and 3 (20% identity, high coverage) Pgbdlike from peptidase (1LBU, 30% identity)

(table continues)

No	Endolysins	Genbank/pham Accession no.	Phage Cluster	Subcluster	AA length	Mass (kDa)	Predicted Function (by SEA-PHAGES)	Notes
**Amidase-X**			**1.2%**					
1	>Kardashian gp4	QEQ93869.1	BI	BI6	286	31.7	Lysin A	26% XylA (3HMA), 38% incomplete CW-7 (5I8L) but CW-7 doesn't align very well.
2	>SendItCS gp4	AWN06107.1	BI	BI4	280	30.9	N/A	23% amidase (AmpDh3 4BXD), 40% incomplete CW-7 (5I8L)
3	>Rainydai gp4	AWN05870.1	BI	BI4	280	30.9	N/A	see SendItCS gp4
**Amidase-Transglycosylase**			**2.0%**					
1	>Warpy gp48	ASN73122.1	BE	BE1	472	50.4	LysM-like PGBD	See evy gp45
2	>Evy gp45	YP_010103421.1	BE	BE1	473	50.5	LysM-like PGBD	27% amidase (4BOL), 32% transglycosylase active site (1GBS)
3	>Jay2Jay gp49	AIW02547.1	BE	BE1	474	50.8	LysM-like PGBD	See evy gp45
4	>Circinus gp19	QBZ72303.1	BK	BK2	416	44.6	LysM-like endolysin	
5	>BillNye gp17	YP_009622595.1	BK	BK2	418	44.7	LysM-like endolysin	
**CHAP-LysM**			**0.4%**					
1	>Sros11 gp23	pham 97340	BA	None	310	33.0	N/A	
**CHAP-PG-bd-like**			**13.2%**					
1	>phiC31 gp50	CAA07120.2	BB	BB1	362	38.4	N/A	26% to C-ter of 4HPE, PG-bd-like 30%to 5TV7
2	>phiBT1 gp50	CAD80117.1	BB	BB1	278	29.8	N/A	
3	>Vash gp19	QAX93275.1	BB	BB1	275	29.2	lysin A	
4	>Lilbooboo gp19	YP_009819898.1	BB	BB1	273	29.0	lysin A	See Vash
5	>Euratis gp19	QAX94014.1	BB	BB1	275	29.5	lysin A	20-23% CHAP (4HPE and 5UDM), 28% PG-bd-like (5TV7)
6	>Shawty gp19	QAY26944.1	BB	BB1	280	29.8	lysin A	
7	>TG1 gp19	AFU62214.1	BB	BB1	276	29.8	putative endolysin	
8	>RemusLoopin gp19	QBZ73333.1	BB	BB2	270	29.4	lysin A	
9	>Heather gp19	QBZ73389.1	BB	BB2	279	31.3	lysin A	
10	>Sebastisaurus gp19	QAX95007.1	BB	BB2	275	29.5	lysin A	
11	>Thestral gp27	YP_010055810.1	BD	BD2	363	39.0	Lysin A	
12	>Omar gp27	AUG87211.1	BD	BD2	286	31.2	Lysin	
13	>TinaBelcher gp26	QAY15849.	BD	BD2	363	39.1	Lysin A	
14	>Bowden gp27	QAY15685.1	BD	BD2	369	39.6	Lysin A	

15	>Alvy gp28	YP_010055722.1	BD	BD2	370	40.0	Lysin A	
16	>Daudau gp26	YP_010055638.1	BD	BD2	280	29.8	Lysin	

(table continues)

No	Endolysins	Genbank/pham Accession no.	Phage Cluster	Subcluster	AA length	Mass (kDa)	Predicted Function (by SEA-PHAGES)	Notes
**CHAP-PG-bd-like**			**13.2%**					
17	>TrvxScott gp25	AXQ62351.1	BD	BD2	368	39.3	Lysin A	
18	>BartholomewSD gp28	QAX95478.1	BD	BD2	370	40.0	Lysin A	
19	>Caelum gp27	YP_010055476.1	BD	BD2	285	30.8	Lysin A	
20	>Alsaber gp28	YP_010056359.1	BD	BD3	285	31.0	N/A	
21	>Saftant gp27	YP_010056207.1	BD	BD3	288	31.0	Lysin A	
22	>StrepC gp26	pham 97340	BD	BD5	276	30.1	N/A	
23	>Araceli gp35	QFG07849.1	BH	None	248	26.8	endolysin	
24	>Intolerant gp34	QFG07929.1	BH	None	252	27.1	endolysin	
25	>Microdon gp34	AYD86781.1	BH	None	252	27.1	LysM-like endolysin	
26	>LazerLemon gp35	AWY07518.1	BH	None	252	27.5	endolysin	
27	>Crosby gp35	AXH49423.1	BH	None	252	27.2	lysin A	
28	>UNTPL gp35	AWY07436.1	BH	None	252	27.0	endolysin	
29	>Henoccus gp35	AWY07273.1	BH	None	250	26.9	endolysin	
30	>JackieB gp34	AWY07353.1	BH	None	248	26.8	endolysin	
31	>Nesbitt gp21	AVO22278.1	BL	None	284	30.6	lysin	
32	>Rowa gp21	YP_009796855.1	BL	None	284	30.1	lysin	
33	>AbbeyMikolon gp21	YP_009796743.1	BL	None	277	29.8	lysin	
**Zinc peptidase-CW-7**			**10.8%**					
1	>FrodoSwaggins gp4	QGJ96544.1	BI	BI1	291	32.4	endolysin	
2	>FidgetOrca gp5	QGJ96704.1	BI	BI1	289	32.2	endolysin	
3	>Meibysrarus gp5	QEQ93953.1	BI	BI1	289	32.2	endolysin	
4	>Jaylociraptor gp5	QEQ93698.1	BI	BI1	289	32.2	endolysin	
5	>Hoshi gp5	QEQ94222.1	BI	BI1	289	32.2	endolysin	
6	>GirlPower gp4	QEQ93503.1	BI	BI1	288	32.0	endolysin	
7	>CherryBlossom gp5	QEQ93784.1	BI	BI1	289	32.0	endolysin	
8	>Esketit gp5	QDM56506.1	BI	BI1	289	32.2	lysin A	
9	>Soshi gp4	QEQ94617.1	BI	BI1	291	32.4	endolysin	

10	>Popy gp4	QAY17038.1	BI	BI1	291	32.4	endolysin	
11	>Namo gp5	QAY16303.1	BI	BI1	289	32.2	endolysin	

(table continues)

No	Endolysins	Genbank/pham Accession no.	Phage Cluster	Subcluster	AA length	Mass (kDa)	Predicted Function (by SEA-PHAGES)	Notes
**Zinc peptidase-CW-7**			**10.8%**					
12	>Madamato gp5	QAY17123.1	BI	BI1	289	32.1	Endolysin	
13	>Spectropatronm gp5	ASU04001.1	BI	BI1	289	32.2	endolysin	
14	>DrGrey gp4	YP_009612534.1	BI	BI1	291	32.4	endolysin	
15	>IceWarrior gp5	QAY16217.1	BI	BI1	289	32.2	endolysin	
16	>Rima gp5	AOZ64959.1	BI	BI1	289	32.2	endolysin	
17	>OlympicHelado gp5	AOZ64870.1	BI	BI1	289	32.2	endolysin	
18	>Maya gp5	QNN98170.1	BI	BI1	289	32.2	endolysin	
19	>TieDye gp4	QNN99480.1	BI	BI1	289	32.2	endolysin	
20	>PherryCruz gp3	QBZ73430.1	BI	BI2	294	32.9	N/A	
21	>RavenPuff gp3	AWN05960.1	BI	BI2	294	32.9	N/A	
22	>Moozy gp4	AWN05439.1	BI	BI2	294	32.8	N/A	
23	>HotFries gp3	AWN05171.1	BI	BI2	294	32.8	N/A	
24	>Scap1 gp4	ATN93653.1	BI	BI2	289	32.2	endolysin	
25	>LibertyBell gp4	AXQ61245.1	BI	BI3	279	31.4	endolysin	
26	>Bing gp4	YP_009622802.1	BI	BI5	290	32.1	endolysin	
27	>RosaAsantewaa gp4	QBZ73568.1	Singleton	None	287	32.0	N/A	
**Glycosyl hydrolase-SH3**			**0.4%**					
1	>Shyg gp25	pham 4657	BC	BC4	304	32.6	N/A	
**Transglycosylase (SAR)**			**12.4%**					
1	>Evy gp40	YP_010103416.1	BE	BE1	174	19.0	Endolysin	
2	>Daubenski gp41	YP_010104804.1	BE	BE1	178	19.2	Endolysin	
3	>Braelyn gp37	YP_010103929.1	BE	BE1	179	19.8	Endolysin	See NootNoot
4	>Teutsch gp39	QAX95775.1	BE	BE1	182	19.7	Endolysin	
5	>Egole gp40	YP_010101461.1	BE	BE1	184	19.8	Endolysin	
6	>Tribute gp38	QGH78230.1	BE	BE1	182	19.7	Endolysin	
7	>Mildred21 gp38	YP_009610579.1	BE	BE1	197	21.6	Endolysin	24% transglycosylase (1GSA, 1SLY), no N-term predicted
8	>NootNoot gp34	YP_009610809.1	BE	BE1	179	19.7	Endolysin	
9	>Paradiddles gp34	YP_009611030.1	BE	BE1	179	19.8	Endolysin	see NootNoot

10	>Warpy gp42	ASN73116.1	BE	BE1	178	19.4	Endolysin	See Tribute
11	>Sushi23 gp40	ASR76472.1	BE	BE1	183	19.6	Endolysin	see Teutsch gp39

(table continues)

No	Endolysins	Genbank/pham Accession no.	Phage Cluster	Subcluster	AA length	Mass (kDa)	Predicted Function (by SEA-PHAGES)	Notes
**Transglycosylase (SAR)**			**12.4%**					
12	>Samisti12 gp39	YP_009611477.1	BE	BE1	182	19.7	Endolysin	See Peebs
13	>Peebs gp39	YP_009611251.1	BE	BE1	182	19.7	Endolysin	See Egole
14	>Jay2Jay gp42	AIW02540.1	BE	BE1	178	19.4	Endolysin	See Warpy
15	>MulchMansion gp37	QNO12461.1	BE	BE1	178	19.2	Hydrolase	
16	>LilMartin gp37	QNN98287.1	BE	BE1	178	19.2	N/A	
17	>Bmoc gp37	YP_010107437.1	BE	BE1	175	18.8	endolysin	21% transglycosylase (4HPE,4OW1, 6CFC), no N-term predicted
18	>IchabodCrane gp38	QFP97354.1	BE	BE2	181	20.1	Endolysin	See Karimac
19	>Bordeaux gp39	QGH79811.1	BE	BE2	181	20.0	Endolysin	See Karimac
20	>Genie2 gp41	QAY08702.1	BE	BE2	181	20.2	Endolysin	
21	>BoomerJR gp41	QAY12692.1	BE	BE2	181	20.2	Endolysin	See Yaboi
22	>Yaboi gp41	YP_009841177.1	BE	BE2	181	20.2	Endolysin	See Karimac
23	>Wipeout gp38	QGH74284.1	BE	BE2	181	20.0	Endolysin	See Karimac
24	>TomSawyer gp39	QGH78926.1	BE	BE2	181	20.0	Endolysin	See Karimac
25	>Wofford gp39	YP_009839727.1	BE	BE2	184	20.3	Endolysin	29% transglycosylase (1GSA), no N-term predicted.
26	>Starbow gp39	AXH66548.1	BE	BE2	181	20.1	Endolysin	See Karimac
27	>LukeCage gp40	YP_009839964.1	BE	BE2	181	20.2	Endolysin	See Karimac
28	>StarPlatinum gp40	YP_009839477.1	BE	BE2	181	20.1	Endolysin	See Karimac
29	>Birchlyn gp38	QDF17214.1	BE	BE2	181	20.0	Endolysin	See Karimac
30	>Karimac gp40	YP_009840212.1	BE	BE2	181	20.1	Endolysin	
31	>MindFlayer gp39	QPL13679.1	BE	BE2	181	20.1	Hydrolase	See Karimac
